# Utility of objective gait measures in levodopa‐unresponsive freezing in Parkinson's

**DOI:** 10.1002/ccr3.3640

**Published:** 2021-01-13

**Authors:** Tuhin Virmani, Aliyah Glover, Lakshmi Pillai, Mitesh Lotia, Rohit Dhall

**Affiliations:** ^1^ Department of Neurology University of Arkansas for Medical Sciences Little Rock AR USA; ^2^ Center for Translational Neuroscience University of Arkansas for Medical Sciences Little Rock AR USA

**Keywords:** falls, freezing of gait, gait, levodopa‐unresponsive freezing of gait, Parkinson's disease

## Abstract

Levodopa‐unresponsive gait freezing in Parkinson disease is debilitating. Gait kinematics, while time‐consuming, can help optimize levodopa's benefit on gait stride length and stride velocity, and thereby improve freezing and falls in these patients.

## Graphical abstract

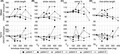

## 1 INTRODUCTION

Levodopa‐unresponsive gait freezing is a debilitating feature of Parkinson's disease for which treatments are limited. Six patients underwent gait evaluation both OFF and ON 3 different levodopa doses. Three different dose titration curves were identified. At optimized dose, 3/5 patients had improved FOG‐Q scores, and 4/5 reduced fall frequency.

Freezing of gait (FOG) manifests as the sensation of the feet “sticking to the ground” during initiation of movement or during active movement.^1^ FOG is one of the more debilitating motor complications of Parkinson's disease (PD),^1^ as it can lead to falls,^2^, ^3^ development of the fear of falling,^4^ and decreased quality of life.^5^ FOG typically is levodopa responsive earlier in the disease course. As disease progresses, some patients develop levodopa‐unresponsive freezing of gait (ON‐state freezing), in which higher levodopa doses may not improve freezing and may in fact worsen the severity of gait freezing.^6^ Symptom diaries and questionnaires^7^, ^8^ have been used to quantify frequency and severity of FOG, but are inherently subjective and made less reliable by concurrent cognitive impairments in PD patients. This makes clinical dose adjustment very difficult in these patients.

Levodopa does help improve gait clinically with increased stride length, velocity, and reduced shuffling of the feet. However, during clinic visits, gait is subjectively assessed by trained movement disorders neurologists watching a patient walk up and down a short hall every few months between dose adjustments. This makes it difficult to determine the best dosage of levodopa in patients reporting variable subjective responses to medication titration at home.

Gait kinematics is clearly affected in PD patients with levodopa responsive freezing of gait.^9^, ^10^, ^11^, ^12^, ^13^, ^14^ Our objective in this study was to utilize a select number of spatiotemporal gait parameters that provide information on the salient features of gait and to determine their utility in gait optimization in patients with levodopa‐unresponsive freezing of gait.

## 2 MATERIALS AND METHODS

Patients with PD based on UK brain bank diagnostic criteria,^15^ and levodopa‐unresponsive freezing of gait (FOG) that did not improve despite attempts to clinically optimize levodopa dosing, were asked to participate. All patients had provided written informed consent to participate in a longitudinal study monitoring gait progression in PD (UAMS IRB# 203 234). This protocol included gait monitoring with clinical levodopa adjustments in the OFF levodopa and ON levodopa state. The study was conducted in accordance with the guidelines of the Declaration of Helsinki.

Patients presented to the gait laboratory in the Movement disorders clinic at the University of Arkansas for Medical Science in the morning in the levodopa OFF medication state on three occasions over a maximum period of 10 days to limit disease fluctuations. All patients underwent an objective gait assessment in the levodopa OFF state and then again 60 minutes after different doses of levodopa on different days. The range of dose administered was individualized to the patient based on their prior home dose. Gait was assessed at the first visit in OFF state and then again following their home levodopa dose amount. At the second visit, after OFF state assessment, the levodopa dose administered was adjusted downward by 50‐100 mg depending on their individual absolute dose. The third dose was chosen based on the direction of change in stride length observed between the first two doses. If a longer stride length was noted at the second visit, the third dose tested was lowered further. If a shorter stride length was noted at the second visit, the third dose tested was increased. The lowest levodopa dose assessed was 100 mg.

For gait assessments, patients were instructed to walk at a “comfortable” pace, 8 lengths of a 20′ × 4′ Zeno Walkway pressure sensor impregnated mat (Protokinetics). Pressure sensor data were collected and analyzed using PKMAS software (Protokinetics). Freezing episodes were excluded from the analysis. The mean and stride‐to‐stride variability (measured as the percent coefficient of variability (%CV)) for steady state gait for each patient at each visit in both levodopa OFF and ON conditions was calculated for 4 spatiotemporal parameters of gait. Stride length (SL) was chosen as it has previously been reported decreased in freezers compared to nonfreezers.^10^, ^11^, ^16^ Additionally, the successive reduction in stride length, in the setting of an already reduced stride length (termed the sequence effect), has been suggested to provoke freezing of gait.^10^, ^12^, ^17^ Stride velocity (SV) was chosen as slowing is a core feature of PD. Swing phase percent (Sw%) was chosen as decreased time spent in this phase of the gait cycle would suggest greater shuffling of gait. Finally, foot‐strike length (FL) was chosen as we have previously reported that freezers have increased variability in foot strike compared to nonfreezers.^11^ Levodopa response for each spatiotemporal gait parameter was calculated as ON/OFF levodopa, using each mornings OFF levodopa examination to obtain the response for that particular dose, (ie, ON 100 mg/OFF 100 mg same day) thereby reducing effects from any day‐to‐day fluctuations. The most recent Montreal Cognitive Assessment Score (MoCA)^18^ from their study visits is also reported. Patients also were scored on the freezing of gait questionnaire (FOG‐Q),^7^ their estimated fall frequency was calculated, and their levodopa response on the motor Unified Parkinson Disease rating scale score (UPDRS)^19^ was calculated (on their pretitration dose).

## 3 RESULTS

The demographics of our 6 PD patients are shown in Table 1. The age of patients ranged from 70 to 77 years old. All patients reported daily freezing of gait that did not improve with their levodopa dose and/or worsened after their levodopa dose (levodopa‐unresponsive or ON‐state freezing). All had a history of falls from gait freezing and were using walkers to help prevent falls (Hoehn and Yahr Stage 4). The duration of freezing of gait (1.7‐4.5 years) was quite heterogeneous and not necessarily related to disease duration (4.4‐13.8 years) or levodopa dose (range 300‐1900 mg/day). Patient 3, who had the longest disease duration (13.8 years), had one of the shortest durations of reported freezing (1.7 years) and had been on levodopa the longest (9.8 years). ON state freezing occurred at very different levodopa doses as Patient 1 was tested on an outpatient basis between the range of 50 and 150 mg/dose while Patient 4 between 200 and 500 mg/dose.

**TABLE 1:** Patient characteristics

	Patient 1	Patient 2	Patient 3	Patient 4	Patient 5	Patient 6
Age at visit (y)	73.0	71.5	71.6	77	76.9	70.0
Gender	Male	Male	Male	Male	Male	Female
Disease duration (y)	5.5	5.7	13.8	8.1	14.2	9.3
MoCA score	25	24	17	26	16	24
FOG duration (y)	3.5	2.6	1.7	4.5	1.6	3.1
Motor UPDRS score on/off (% improvement)	37/41.5 (11%)	21/33.5 (37%)	16/29 (45%)	31.5/33 (5%)	35/40.5 (14%)	18/26.5 (32%)
ON state total UPDRS score	62	40	37	56.5	56.5	34
On agonist	No	Ropinirole 3 mg	No	No	No	No
On MAO‐I	No	Rasagiline 1 mg	Selegiline 5 mg	No	No	No
Duration on levodopa (y)	2.3	5.7	9.8	5.1	4.3	5.0
Recent levodopa range/day	150‐450 mg	400‐800 mg	1340‐2140 mg	1600‐1900 mg	750‐1250 mg	400‐1000 mg
Recent levodopa range/dose	50‐150 mg	100‐200 mg	150‐270 mg	200‐500 mg	250‐400 mg	100‐250 mg
Pretitration levodopa daily dose	300 mg	800 mg	1490 mg	1900 mg	1000 mg	1000 mg
Pretitration levodopa single dose	100 mg	200 mg	150‐270 mg^a^, ^b^	200‐500 mg^a^	250‐400 mg^a^	250 mg
Optimized levodopa single dose	100 mg	100 mg	200 mg	200 mg	300 mg	100 mg
Levodopa daily dose at 3 mo follow‐up	300 mg	400 mg	1220 mg	(target 1000 mg)	1000 mg	650 mg
FOG‐Q total score/subscore (Q3‐6): Pretitration	15/11	15/9	18/13	15/9	15/10	16/11
FOG‐Q score/subscore: 3 mo follow‐up visit	18/13	12/8	16/11	n/a	17/11	16/11
Estimated fall frequency per month: pretitration	0.33	12	12	0.33	1	28
Estimated fall frequency per month: 3 mo follow‐up	0	0	1.5	n/a	4	8

^a^ Taking different doses at different times of the day.

^b^ Taking IR and CR levodopa combinations.

**FIGURE 1:**
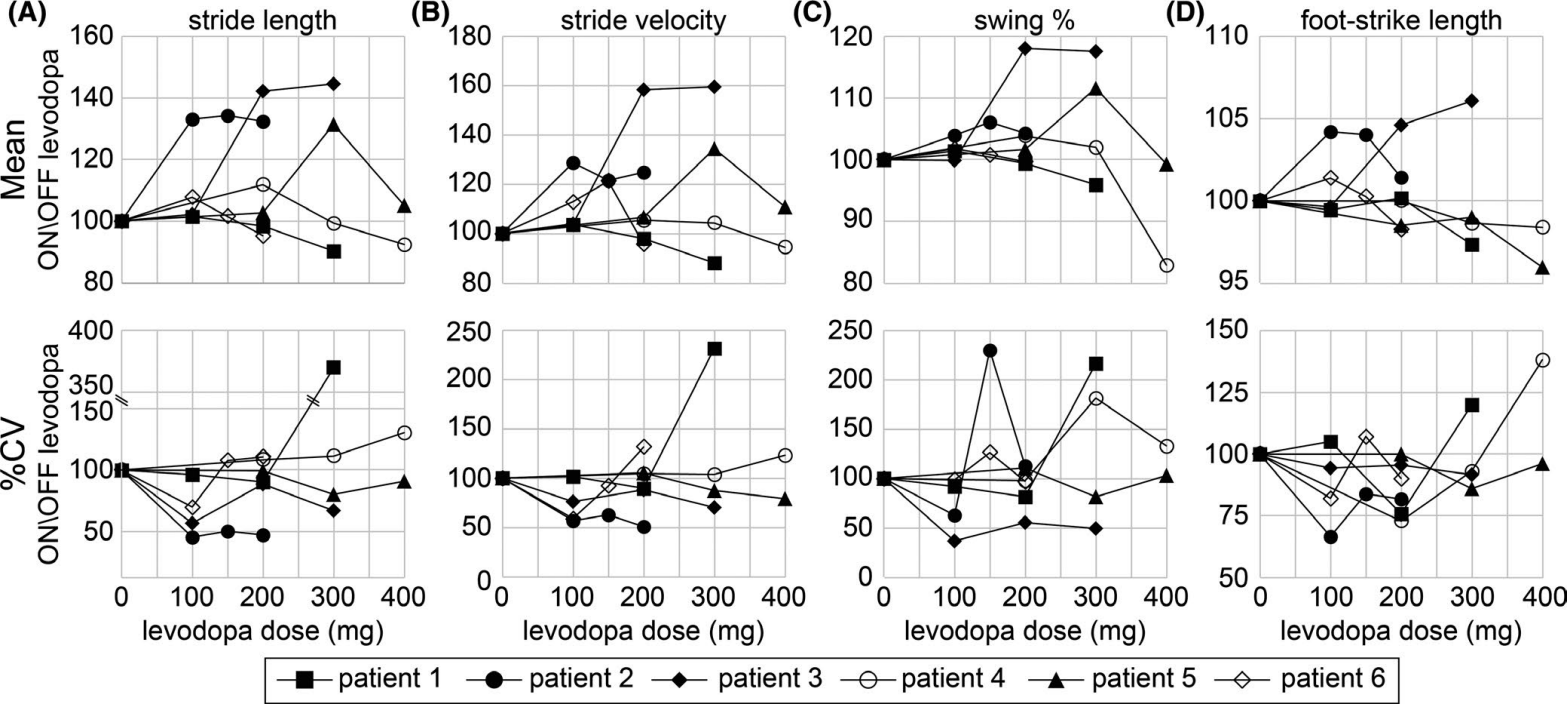
Response of spatiotemporal gait parameters to levodopa titration. Patient specific responses ON levodopa/OFF levodopa in mean (A‐D) and stride‐stride variability (E‐F) and objectively measured spatiotemporal gait parameters

The dose dependent changes in the mean and stride‐stride variability (%CV) in SL, SV, Sw%, and FL are shown in Figure 1. Despite all patients reporting ON‐levodopa freezing of gait, different patients had different response to levodopa dose changes. Patient 2 (filled circle) and Patient 3 (filled diamond) had improved SL (Figure 1A) and SV (Figure 1B; >100% ON/OFF levodopa) that plateaued at 100 mg and 200 mg, respectively. Patient 4 (open circle), 5 (filled triangle), and 6 (open diamond) showed initial improvement at differing doses (200 mg, 300 mg, and 100 mg, respectively) and then subsequently showed a decline. Patient 4 and 6 showed decreased SL and SV ON levodopa that was even worse than their OFF levodopa examinations (<100% ON/OFF levodopa). Patient 1 (filled square) had minimal improvement in SL and SV and declined at higher doses. Sw% (Figure 1C) and mean FL (Figure 1D; measures length of foot strike with the ground) mostly followed changes in SL and SV except in Patient 2 where FL had begun to shorten at 200 mg dose, while SL remained stable and improved. Stride‐to‐stride variability in SL (Figure 1E) and SV (Figure 1F) mostly followed the mean changes with decreased variability (ON/OFF levodopa < 100%) corresponding to improved mean variables. Variability in Sw% (Figure 1G) and FL (Figure 1H) was not as consistent with varying levodopa doses. For example, Patient 2 had improved mean Sw% but worsened %CV Sw% at 150 mg levodopa. Patient 1 had no clear change in mean FL at 200 mg although %CV was reduced.

Based on these titration curves, primarily utilizing mean SL and SV, the amount of levodopa taken per dose was reduced in 5 patients and remained the same in 1 patient. At a 3‐month follow‐up visit, completed by 5/6 patients, 3/5 patients had subjective improvements in FOG‐Q scores, 1 was unchanged, and patient 1 who remained on the same levodopa dose had a worsening in FOG‐Q score. Fall frequency was also reduced in 4/5 patients at follow‐up but increased in 1/5.

## 4 DISCUSSION

Levodopa‐unresponsive (or ON‐state) freezing of gait in Parkinson's disease is difficult to treat. Often patients are unable to provide consistent reports on whether levodopa dosage titrations help or worsen their gait and balance at home. In this report, we used spatiotemporal gait parameters, primarily stride length and stride velocity, to optimize levodopa dosing in 6 patients with moderate‐severe ON‐state (levodopa‐unresponsive) freezing of gait. 5/6 patients ended up on lower single administration levodopa doses than they had been taking at home, and subjectively, 3/5 had improved freezing based on FOG‐Q, and 4/5 improved self‐reported fall frequency.

As we studied the dose‐response of patients to levodopa more objectively, the heterogeneity of gait dynamics in freezers became apparent. While all 6 patients had ON‐state freezing, there were three different patterns of changes seen in the mean spatiotemporal parameters of continuous gait with levodopa titration: 1) improved gait at a particular dose and then plateau in improvement with increased dose, 2) initial gait improvement followed by decline at higher doses, and 3) minimal improvement with subsequent decline at higher doses. Stride‐stride variability was even more heterogeneous. This is in comparison with the simple dose‐response curve seen for levodopa on UPDRS scores in the ELLDOPA trial.^20^ As we cannot clinically determine which patient will have a particular levodopa dose‐response curve, integrating objective assessments into clinical care can help individualize therapy. It can also help decrease medication burden as 5 of our patients ended up on lower doses of levodopa.

In summary, in cases of difficult to control, levodopa‐unresponsive (or ON‐state) freezing of gait, levodopa titration using objectively measured spatiotemporal gait parameters can provide clinical utility. The time required to perform these assessments limits more wide spread application, however, and studies with larger cohorts are needed to determine the minimum set of assessments indicated. Based on our results, we have incorporated a smaller footprint instrumented gait mat into our movement disorders clinic (Protokinetics 10′ × 2′ Zeno‐walkway). In our study, we also utilized subjective assessments of freezing severity at home. However, ongoing work validating some of these measures with cheaper wearable devices^21^, ^22^, ^23^, ^24^ may eventually provide more widely deployable, real‐time, home‐based measures of freezing. Therefore, we suggest that the introduction of objective gait assessments into our clinical care algorithms should become standard of care in the future.

## CONFLICT OF INTEREST

Dr Virmani, Ms Glover, and Ms Pillai received salary support from the University of Arkansas Clinician Scientist Program. Dr Virmani, Lotia, and Dhall received salary support from the University of Arkansas for Medical Sciences. None of the other authors have any financial disclosures or conflicts of interest related to the research covered in this manuscript.

## AUTHOR CONTRIBUTIONS

LP and AG: were involved in research project organization and execution and manuscript review and critique. ML and RD: were involved in manuscript review and critique. TV: was involved in research project conception, organization and execution, and writing and revision of the manuscript.

## ETHICAL APPROVAL

All patients had provided written informed consent to participate in a longitudinal study monitoring gait progression in PD (UAMS IRB# 203234). This protocol included gait monitoring with clinical levodopa adjustments in the OFF levodopa and ON levodopa state. The study was conducted in accordance with the guidelines of the Declaration of Helsinki.

## FINANCIAL DISCLOSURES

Tuhin Virmani received salary and grant support from the University of Arkansas for Medical Sciences Clinician Scientist program, NIGMS IDeA Program Center of Excellence award (P30 GM110702) and the Parkinson's Foundation (PF‐JFA‐1935). The author reports no financial interests. Aliyah Glover received salary support from the Clinician Scientist program grant to Tuhin Virmani. The author reports no other financial interests. Lakshmi Pillai received salary support from the Clinician Scientist program grant to Tuhin Virmani. The author reports no other financial interests. Mitesh Lotia received salary support from the University of Arkansas for Medical Sciences. He has also received Clinical Trials support from Pharma‐2B, Neurocrine, Impax Pharmaceuticals, CALA Health, Global Kinetics Corp, and Axovant. Rohit Dhall received salary support from the University of Arkansas for Medical Sciecnes. He has also received Clinical Trials support from Pharma‐2B, Impax Pharmaceuticals, CALA Health, Global Kinetics Corp, and Axovant.

## Data Availability

As these are data from part of an ongoing longitudinal study with continuous enrollment and data collection, our IRB limits public release. Anonymized data sets can be shared at the request of any qualified investigator.
